# Adaptive Immunity to Hepatitis C Virus

**DOI:** 10.3390/v1020276

**Published:** 2009-09-08

**Authors:** Mirjam B. Zeisel, Samira Fafi-Kremer, Eric Robinet, François Habersetzer, Thomas F. Baumert, Françoise Stoll-Keller

**Affiliations:** 1 Inserm, U748, 3 rue Koeberlé, F-67000 Strasbourg, France; E-Mails: mirjam.zeisel@unistra.fr (M.B.Z.); samira.fafi-kremer@chru-strasbourg.fr (S.F.-K.); e.robinet@unistra.fr (E.R.); francois.habersetzer@chru-strasbourg.fr (F.H.); thomas.baumert@unistra.fr (T.F.B.); 2 Institut de Virologie, Université de Strasbourg, 3 rue Koeberlé, F-67000 Strasbourg, France; 3 Pôle Hépato-Digestif, Nouvel Hôpital Civil, Hôpitaux Universitaires de Strasbourg, 1 Place de l’Hôpital, F-67000 Strasbourg, France

**Keywords:** viral clearance, neutralizing antibodies, T cells, escape, vaccine development

## Abstract

The precise role of adaptive immune responses in the clinical outcome of HCV infection is still only partially defined. Recent studies suggest that viral-host cell interactions during the acute phase of infection are essential for viral clearance or progression into chronic HCV infection. This review focuses on different aspects of the adaptive immune responses as determinants of the different outcomes of HCV infection, clearance or persistent infection, and outlines current concepts of HCV evasion strategies. Unravelling these important mechanisms of virus-host interaction will contribute to the development of novel strategies to prevent and control HCV infection.

## 1. Introduction

Hepatitis C virus (HCV), a member of the *Flaviridae* family, infects 3% of the population resulting in chronic infection in the majority of cases. HCV chronic hepatitis frequently results in progressive fibrosis, cirrhosis with an increased risk of hepatocellular carcinoma [1]. These latter complications have become leading indications for liver transplantation in developed countries. There is no vaccine and the standard of care treatment, a combination of pegylated interferon and ribavirin, is limited by resistance in a large fraction of patients, toxicity and high costs. After exposure to HCV, 60 to 80% of infected persons develop persistent viremia despite the generation of HCV-specific antibodies and HCV-specific cellular immune responses [2,3]. Persistent viremia - detected by polymerase chain reaction - remains positive after more than 6 months. Studies of host responses in the course of HCV infection have been hampered by the fact that acute HCV infection is asymptomatic in most individuals and thus frequently not recognized. Moreover, the chimpanzee is the only immunocompetent animal susceptible to HCV infection and there are major differences between HCV infection in chimpanzees and in humans. Studies of the host’s immune responses in humans thus rely on patient cohorts. Through the availability of serial samples from acute and chronic HCV infected patients, insights into the humoral and cellular immune responses in the course of HCV infection could be gained in the past years.

The present review focuses on different aspects of the adaptive immune responses as determinants of the different outcomes of HCV infection, clearance or persistent infection, and outlines current concepts of HCV evasion strategies.

## 2. The humoral responses to HCV infection

Neutralizing antibodies are generally an important mechanism for control of initial viremia and protection from re-infection in viral infections. However, the role of the humoral immune response in the clearance of HCV infection has been questioned for a long time. While anti-HCV antibodies can easily be detected in the course of HCV infection by commercially available antibody assays approximately 50 to 60 days after HCV infection [4], these tests only attest a humoral immune response to HCV proteins but they do not evaluate the neutralizing ability of these antibodies. The ability of antibodies to neutralize HCV can solely be evaluated using relevant model systems.

Determining the relative role of antibodies in the course of HCV infection has long been hampered by the absence of a convenient model system for evaluating the neutralizing activity of anti-HCV antibodies. HCV infects only humans and chimpanzees and for a long time the chimpanzee represented the only validated animal model for the study of HCV (reviewed in [5]). Over the past years, the development of sensitive and robust *in vitro* neutralization assays based on human hepatoma cell lines and HCV pseudotyped particles[6–8], HCV-like particles [9–11] and recombinant cell culture-derived HCV (HCVcc) [12–19] then allowed to conveniently study the role of neutralizing antibodies in acute and chronic HCV infection. Moreover, the recent development of an *in vivo* model based on immunodeficient mice repopulated with human livers, the uPA-SCID mice [20], enabled investigators for the first time to determine the role of antibodies in HCV infection in a small animal model [21,22].

Early studies investigating immune responses in chimpanzees and humans suggested that HCV clearance could occur in the absence of neutralizing antibodies or that antibody responses alone are not sufficient to eradicate HCV in the majority of cases [23–27]. Moreover, individuals who cleared HCV are not protected against re-infection, although chimpanzees and individuals who have cleared HCV seem to be less likely to develop chronic infection after re-exposure [28–30]. Since the development of novel model systems for the study of HCV infection and neutralization *in vitro*, the availability of sequential serum samples from homogenous patient cohorts, well-defined viral inoculum and viral surrogate ligands used for neutralization assays, isolate-specific neutralizing antibodies have been detected in acutely HCV infected individuals who subsequently cleared viral infection. In contrast, the humoral immune responses seem to be delayed in patients developing chronic HCV infection, thereby allowing the virus to escape the host’s immune surveillance.

### 2.1. Neutralizing antibodies and control of viral infection

Since the availability of several *in vitro* HCV model systems [8,9,12–14], considerable progress has been made in understanding how HCV enters into host cells and how antibodies may neutralize this process. Binding and entry of HCV is believed to be a complex process involving both viral and cellular factors. The essential viral factors are the HCV envelope glycoproteins E1 and E2 which have been demonstrated to directly interact with cellular factors and to trigger conformational changes necessary to initiate infection. Several cellular factors have been identified to mediate viral attachment and entry, such as CD81, scavenger receptor class B type I (SR-BI), members of the claudin family and occludin [31–41]. HCV envelope glycoprotein E2 has been demonstrated to directly interact with CD81 and SR-BI [31,32] but the interaction of HCV envelope glycoproteins with the other host entry factors is still elusive [36,38]. As the HCV envelope glycoprotein E1 and E2 interaction with host cell factors is mandatory to initiate productive infection, it is an important target for virus neutralization.

**Figure 1.**
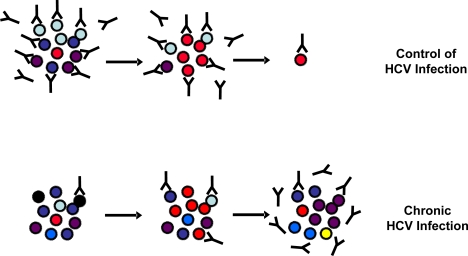
Viral escape from neutralizing antibody responses. In infected individuals, HCV exists as a quasispecies, *i.e.*, a pool of constantly changing, distinct but related genomic variants. Resolving HCV infection is associated with a relatively stable pool of viral variants and the early induction of high-titer cross-neutralizing antibodies. Chronic HCV infection is correlated with diversification of the quasispecies population associated with a delayed induction of cross-neutralizing antibodies allowing viral escape from the host humoral responses.

Using retroviral pseudoparticles bearing HCV envelope glycoproteins (HCVpp), neutralizing antibodies have been detected in patients with acute and chronic HCV infection. The association between the induction of neutralizing antibodies for resolution of infection during acute HCV infection has been demonstrated using well defined viral inoculum and autologous surrogate ligands [42–44]. Lavillette *et al*. and Pestka *et al*. have shown that neutralizing antibodies are induced in the early phase of infection by patients who subsequently control [42] or resolve [43] viral infection. In hemodialysis patients with nosocomial acquired HCV infection, strong neutralizing responses correlated with decrease in viremia and control of HCV replication whereas absent neutralizing response associated with persistent high viremia and failure to control HCV infection [42]. Moreover, in an accidental single-source outbreak of hepatitis C in pregnant women, viral clearance was associated with the rapid induction of high-titer and cross-neutralizing antibodies in the acute phase of infection while chronic HCV infection was characterized by a complete absence or reduced capacity to neutralize the transmitted virus as well as heterologous viruses in the early phase of infection [43]. These results suggest that a strong early broad neutralizing antibody response may contribute to resolution of HCV in the acute phase of infection while delayed induction of neutralizing antibodies may contribute to development of chronic HCV infection (Figure 1).

### 2.2. Viral escape from neutralizing antibodies

As HCV has evolved several mechanisms to escape from the host immune responses (reviewed in [45]), neutralizing antibodies and HCV co-exist during chronic infection in patients who did not mount efficient immune responses able to clear the virus during acute infection (Figure 1). Viral escape from antibody-mediated neutralization has been shown to occur on several levels and in line to reports of other viruses, a combination of different mechanisms may also apply to HCV. These include (1) the high variability of the HCV genome and limited induction of cross-neutralization antibodies, (2) induction of antibodies interfering with neutralizing antibodies, (3) the association of HCV with serum factors such as low-density lipoproteins (LDL) and very low density lipoproteins (VLDL), (4) the interplay of HCV glycoproteins with high-density liporoteins (HDL), (5) the shielding of neutralizing epitopes by glycosylation of defined amino acids of envelope glycoproteins, and (6) direct cell-to-cell transfer of the virus. As these mechanisms have been reviewed elsewhere [45], this review will focus on recent studies demonstrating both *in vitro* and *in vivo* viral adaptations leading to escape from neutralizing antibodies.

Using the state-of-the-art HCV cell culture model, Zhong *et al*. investigated adaptation of HCV *in vitro* [46]. The authors demonstrated that HCV can establish persistent infection *in vitro*, which lead to the selection of viral and cellular variants that favour the survival of both the virus and the host [46]. The virus acquired increased specific infectivity whereas the host cell became resistant to HCV infection. This resistance may be due to down-regulation of HCV entry factor expression or a defect in HCV replication or a combination of these mechanisms [46]. While substantial progress in understanding the HCV life cycle has been made, the interplay between host cell entry factors, HCV envelope glycoproteins and neutralizing antibodies is only about to be investigated. Recent evidence suggests that neutralizing antibodies isolated from chronic HCV patients interfere with entry steps that are closely linked to the interaction of HCV with SR-BI and CD81 [15,47]. Evasion from antibody-mediated neutralization through decreased receptor binding has been reported for viruses such as HIV-1 [48]. This mechanism seems also to apply to HCV. The cell culture-adapted mutation G451R initially described by Zhong *et al*. [46] has been shown to be less dependent on SR-BI and CD81 on the entry level [16]. Moreover, this mutant demonstrated an increased binding to CD81 and CD81 mimics while being more sensitive to neutralizing antibodies [16].

In chronic HCV infected patients, HCV coexists with anti-HCV antibodies. It is thus most interesting to understand how HCV evolves in the presence of neutralizing antibodies. A recent study addressed this important question by investigating *in vitro* HCV escape mutants through multiple rounds of selection by the well-described anti-E2 monoclonal antibody AP33 [49]. The authors described an *in vitro* escape mutation HCV N415Y that lowered viral fitness probably by affecting viral entry but without affecting binding to CD81 [49] suggesting that mutations modulating interaction with host cell factors other than CD81 may contribute to escape of HCV from neutralizing antibodies. Taken together, these studies show that in cell culture, mutations within the HCV envelope glycoproteins arise that modulate viral entry and neutralization by anti-HCV antibodies.

As described above, resolution of infection appears to require rapid, vigourous and multi-specific antiviral host immune responses [43,45,50,51]. Patients who subsequently develop chronic infection have been shown to develop a delayed and inefficient neutralizing antibody response [43] allowing HCV infection to persist for lifetime despite the presence of neutralizing antibodies. It is believed that the adaptive immune system exerts constant pressure on the virus thereby leading to the emergence of HCV escape mutants. However, little is known about the role of neutralizing antibodies in driving HCV sequence evolution in the course of infection. A recent study addressed this important question and provided insights into the time-course of induction of neutralizing antibodies and viral escape from neutralizing responses in a cohort of young intravenous drug users [44]. Studying autologous humoral immune responses in individual subjects, the authors demonstrate that during acute HCV infection, earlier HCV variants were neutralized by autologous plasma samples prior to neutralization of later HCV variants, similar to what has been shown in a chronic HCV patient [52], suggesting that neutralizing antibodies are responsible for envelope sequence changes over time [44]. In line with previous results obtained in a cohort of patients from a single-source HCV outbreak [43], this study demonstrated an association of high-titer neutralizing antibodies and spontaneous viral clearance whereas persistent HCV infection was associated with low-titer or absent neutralizing antibodies during the acute phase [44]. These data suggest that humoral immune response pressure drives HCV envelope glycoprotein sequence evolution resulting either in effective clearance of circulating viral variants and resolution of infection or emergence of viral escape variants and progression into chronic infection. Analysis of sequence substitutions that occurred in HCV envelope glycoproteins during acute infection were monitored throughout E1E2 but most of them were located in the HVR1 region [44]. Mapping of amino acid substitutions involved in escape from neutralizing antibodies showed that significant loss of sensitivity to neutralizing antibodies could be attributed to 3 HVR1 mutations (K384T, K408R and S405P) [44]. A similar time-course study had previously been conducted in the well-characterized chronic HCV patient H [52]. Consistent with the results obtained during the acute phase study described above [44], von Hahn *et al*. demonstrated that throughout the course of this chronic HCV infection, the patient’s antibodies lagged behind the rapidly evolving viral variants, *i.e.* they were able to neutralize HCV strains that had been circulating several months or years before but not the present or future viral variants of the patient [47,52]. This raised the question of the mechanisms underlying escape of such quasispecies from neutralizing antibodies. By investigating the interaction of these neutralizing antibody-escape variants and HCV host cell factors, Keck *et al*. described a single viral variant from this patient that was characterized by reduced infectivity, diminished CD81 binding and resistance to a panel of anti-E2 antibodies (domain B antibodies and AP33). Thus by escaping from neutralizing antibodies, HCV seems to loose in infectivity due to lower binding to CD81. It is worth noting that several mutations within E2 but outside the anti-E2 epitopes as well as the CD81 binding regions may account for escape from these neutralizing antibodies as site-directed mutations were able to restore sensitivity to neutralizing antibodies and CD81 dependency [47]. The most important mutations responsible for reduced infectivity and binding to CD81 were S501N and V506A, suggesting that mutation of theses amino acids affect the conformation of E2 necessary for interaction with CD81 [47]. However, these mutations did not account for escape from humoral responses. Interestingly, an additional mutation at residue 444 is necessary in order to lead to complete escape from neutralizing antibodies – this additional mutation at position 444 seems to negatively modulate antibody-mediated neutralization in concert with the mutations at residues 501 and 506 [47].

Recently, Zhang *et al*. described an additional escape mechanism whereby the presence of non neutralizing antibodies interferes with the function of neutralizing antibodies, resulting in the reduction or blockage of their effect [53,54]. Two epitopes within a short segment of E2 were mapped: epitope I, at amino acids 412–419, and epitope II, at amino acids 434–446. Epitope I has been recognized as an important neutralization site, while epitope II interfered with antibody to epitope I inhibiting neutralization of the virus [53]. Epitope I- and epitope II- specific antibodies were detected in plasma from chronically HCV-infected patients. Kinetic studies in patient H revealed that antibody to epitope II appeared within 51 days of infection, while antibody to epitope I was not detectable until day 643. Interestingly, by absorbing out antibody to epitope II, neutralizing activity of plasma was enhanced and broadened to include additional genotypes of HCV [54].

## 3. T cell responses to HCV infection

The majority of primary infections are asymptomatic and often unrecognized. Thus, studies of T cell immune responses during acute HCV infection have only been possible in experimentally infected chimpanzees or individuals with occupational needle stick exposure or IVDU involved in epidemiological follow-up for which the time of contamination is documented. A large body of evidence suggests that a strong, multispecific and long-lasting T-cell immune response appears to be important for control of viral infection (reviewed in [27,55]).

Three types of T cell-mediated responses can be raised against HCV [27,55]. First, an efficient primary immune response during the acute phase, leading to a resolved HCV infection and maintenance of an efficient CD4 and CD8 memory. This immune response is sustained and targets multiple viral proteins, especially during the acute phase of the response. Second an efficient but transient primary immune response, leading to partial control of the infection, but ultimately CD4 memory cells are absent while CD8 memory cells are present at a variable level, leading to chronic infection. Third, a lack of efficient primary immune response, leading to chronic infection. Memory CD4 and CD8 memory cells are less frequent, functionally impaired and target less viral proteins than in patients with resolved infection.

This review will focus on cellular and viral factors that may influence the efficiency and maintenance of primary T cell mediated immune responses including incomplete differentiation of effector and memory T cell populations, immune exhaustion resulting from persistent high viral loads mediated by programmed death-1 (PD-1) protein signalling, suppression by regulatory T (Treg) cells and immune escape mutations.

### 3.1. T cell immune responses and control of viral infection

In humans and chimpanzees a self-limited course of acute hepatitis C is associated with vigorous CD4+ and CD8+ T cell responses targeting multiple HCV regions and with intrahepatic production of IFN-γ [23,24,51,56,57]. In a study of five healthcare workers the only subject able to clear acute HCV infection mounted an early, vigorous and sustained CD4+and CD8+ T cell response [51]. A study using IFN-γ enzyme-linked immmunospot (ELISPOT) and human histocompatibility leukocyte antigen (HLA) peptide tetramer assays, revealed that at the earliest time points of acute infection highly activated CTL populations are observed that temporarily fail to secrete IFN- γ, a “stunned” phenotype, from which they recovered as viremia declined [24]. Resolution of acute infection was associated with T cell recovery of an activated phenotype and the ability to produce IFN-γ [24].

The non structural proteins have been described to be preferentially targeted by the CD4 +T cell responses in those who clear infection [58–61]. One of the most recent cross-sectional study of proliferative responses in 22 subjects with resolved infection and 23 with chronic infection showed that at least three of the six non-structural proteins were targeted by all subjects who had cleared HCV infection, with less frequent responses against the core protein and the variable regions of the envelope protein [58]. In all studies, one or more epitopes on NS3 were targeted, suggesting that epitopes in this protein may be immunodominant [50,59,62,63]. This observation is supported by a study examining IFN-γ ELISpot responses to three peptide pools spanning NS3 which showed that all subjects who had recovered from infection mounted a strong CD4+ response to all three pools [62]. Similar to CD4+ T cell responses against HCV, it has been suggested that the breadth of the CD8+ T cell response is associated with clearance in both humans and chimpanzees [23,24,64]. In a recent study of 17 patients with acute HCV leading to persistence and 14 with primary infections resulting in clearance [65] this notion was corroborated: total HCV-specific specific CD4+ and CD8+ T-cell responses were examined and functional T-cell thresholds that predict recovery identified. The likelihood of recovery was considerably greater in individual subjects exceeding these thresholds ; for example if five or more HCV peptides pools (or ∼15% of the HCV genome) are targeted by CD4+ T cells early after infection the chance of recovery was more than seven times higher than if this threshold was not achieved. Similarly it has been shown by logistic regression analysis that patients demonstrating HCV-specific IFN- γ-producing CTL responses to at least two HCV peptides pools were statistically more likely to contain HCV infection than patients demonstrating responses to only one or none of the HCV peptide pools.

The kinetics of onset and the durability of the cellular immune responses may also be an important determinant of outcome. Both human and chimpanzee studies have demonstrated a CD4+ response that is initially effective with a subsequent rebound in viremia and progression to chronic infection [23,51]. A prospective study of 20 subjects with acute infection showed that the number of Th1 cytokine–producing CD4+ cells was higher in the first 12 weeks after disease onset in the subjects with rapid viral clearance compared to those with only transient or no control of viremia [63]. The strongest CD4+ T cell response to HCV infection has been shown to occur within the first six months after infection regardless of outcome [60,63,64,66]. Thus, it appears that a successful CD4+ T cell response needs to develop early and also to be sustained to achieve viral clearance [67].

Smyk-Pearson *et al*. studied the relative importance of CD4 help in spontaneous recovery in acute HCV infection and demonstrated that [65] the presence of HCV-specific cytotoxic T lymphocytes – able to proliferate, exhibit cytotoxicity and produce IFN – γ - did not ensure recovery, but whether these CTLs were primed in the presence or absence of T-cell help (HCV-specific IL-2 production) was a critical determinant. This is also strongly supported by CD4-depletion studies in the chimpanzee model of infection [68]. Helper CD4+ T cells are important through the maintenance of the effector functions of cytotoxic CD8+ T cells. This is mediated both by activation of co-stimulatory pathways and via the production of cytokines notably IL-2 and IFN – γ [69]. Only patients able to finally control infection show maturation of CD8 memory sustained by progressive expansion of CD127+ CD8 cells [67].

The cellular immunity appears to persist for many years after resolution of infection in chimpanzees and humans [62,66,70]. After viral clearance, memory T cells maintain over decades and can mediate protective immunity in spontaneously HCV-recovered chimpanzees following re-challenge with homologous and heterologous HCV [29,30,71]. The rapid control of HCV viremia following re-challenge was found to be associated with early anamnestic HCV-specific CD4+ and CD8+ T cell responses, including memory CD4+ T cell responses [68,72–74]. However, recent studies in chimpanzees contradict the early studies [75]. Thus, a chimpanzee that had previously demonstrated protective immunity following multiple re-challenges with heterologous viruses became chronically infected when re-exposed to the virus originally inoculated into the animal [75]. These findings are supported by evidence that chimpanzees tend to mount weak humoral responses to HCV envelope glycoprotein E2. Indeed, a lower percentage of HCV-inoculated chimpanzees develop detectable antibodies to envelope glycoproteins E1 (22%) and E2 (15%) as compared to humans [76].

### 3.2. Mechanisms of T cell failure

In contrast to acute resolving HCV infection, persisting acute HCV infection is associated with a weak and only monospecific CD4+ T cell responses [77]. Regarding the role of CD8+ T cells, recent studies in humans demonstrated that even strong CD8+ T cell responses in the acute phase of infection may not be adequate to prevent progression to chronicity [64,67,78]. Urbani *et al*. showed that at clinical onset, CD8 responses are similarly weak and narrowly focused in both self-limited and chronically evolving infections [67]. At this stage, CD4 responses are deeply impaired in patients with a chronic outcome as they are weak and of narrow specificity, unlike the strong, broad and T helper 1-oriented CD4 responses associated with resolving infections.

An important issue is to determine what signals allow to sustain memory cells. In murine models of viral infections, an acute viral infection is generally associated with a high expansion of effector cells that differentiate from naïve cells [79]. This expansion phase is followed by a contraction phase leading to the elimination of ∼90% of effector cells, while the remaining effector cells differentiate into long-lived protective memory cells. In human, the differentiation pathways of effector and memory cells may not be similar, and effector cells may be replenished from memory cells. Therefore, it is of crucial importance to identify the mechanisms that allow some patients to maintain HCV-specific memory, while some other are inefficient in controlling the infection. Some key factors may be IL-7 and IL-15, that have been demonstrated to be involved in the induction and homeostasis of CD8 memory cells [80]. IL-7Rα expression is decreased upon T-cell activation: during acute viral infections, the expression of IL-7Rα by viral antigen-specific T cells is transiently decreased [69,81,82] and recovers at late time points after infection when an efficient memory response is obtained [69,83] while IL-7Rα expression remains at a low level in the setting of inefficient memory responses in chronically infected subjects [81]. IL-7Rαhigh expression may therefore allow identifying cells that will give rise to memory cells, at least in the setting of infections that lead to an inflammatory response [69], but not when antigen is presented in a non inflammatory context [84]. Indeed, IL-7Rα expression follows an IL-7-independent program of expression [85] that may be controlled by the level of inflammation [84,85] or the strength of TCR signalling or viral load at time of antigen presentation [82]. IL-15 is also involved in survival of memory cells, especially when IL-7 signalling is present in limiting conditions [69,80]. Indeed, IL-15 is critical for memory cell survival in normal animals, where IL-7 may be limiting due to competition with naïve cells, which use IL-7, but not IL-15, signalling for homeostatic proliferation [86–90]. The CD4-mediated production of another cytokine of the same family, IL-21, has been shown recently to be of crucial importance in avoiding deletion and maintaining memory responses of CD8 T cells in the murine model of LCMV infection [91–93]. Whether IL-21 is also critically involved in maintaining memory HCV-specific memory in humans remains to be determined.

In the context of HCV infection, expression of IL-7Rα by total CD4 and CD8 T cells as well as by HCV-specific cells has been reported to be reduced in the blood of patients with chronic infection as compared with patients with resolved infection [94], although such decreased IL-7Rα expression by HCV-specific memory cells remains controversial [95,96]. IL-7Rα expression is even more decreased in liver CD8+ lymphocytes than in blood lymphocytes from chronic patients [95]. Interestingly, patients with acute infection who subsequently resolved the infection had higher baseline values of IL-7Rα expression (*i.e.* at time of acute infection) than patients with acute infection who subsequently evolved toward chronic infection [94]. However, this picture may be even more complex, as two profiles of IL-7Rα expression have been observed in chronic HCV patients: most patients have exhausted HCV-specific CD8+ T cells, with low IL-7Rα expression, low proliferative and IFN-γ secretion potential, but some patients have HCV-specific T cells that express high levels of IL-7Rα expression and maintain an efficient proliferative and IFN-γ secretion potential, similar to HCV-specific T cells from patients who resolved their infection [96].

**Figure 2.**
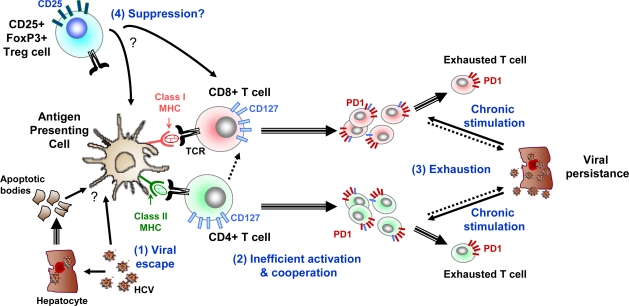
Examples of mechanisms resulting in impairment of T cell responses leading to chronic HCV infection. Chronic HCV infection is associated with impaired CD8+ T cell responses including reduced cytotoxic potential, reduced secretion of Th1 type cytokines and reduced proliferative capacity in response to *ex vivo* antigenic stimulation. Four possible mechanisms of T cell response failure are shown here: (1) viral escape with mutations in HLA restricted epitopes impairing antigen recognition, (2) loss of functional CD4+ T cell responses, (3) overexpression of PD1 in CD8+ T cells; when PD1 binds to its ligand PD-ligand 1 (PD-L1), which is preferentially expressed by virus-infected cells, an inhibitory signal is transmitted to CD8+ T cells, resulting in blocking of the T cell receptor-mediated activation signal, (4) induction of regulatory T cells. Arrows with single line indicate functional interactions while arrows with double lines indicate cell differentiation.

In the chronic phase, virus specific CD4+ and CD8+ T cell responses are also detectable. However HCV-specific CD4+ and CD8+ T cells isolated from chronically infected patients usually display functional and maturation defects including reduced cytotoxic potential, reduced secretion of Th1-type cytokines and a reduced proliferative capacity in response to *ex vivo* antigenic stimulation [57,97,98]. CD8+ T cell exhaustion such as observed during chronic HCV infection is described in different murine models of persistent infection with highly replicative viruses and may result from deficient CD4+ T cell help (reviewed in [99]). Ulsenheimer *et al*. have described functionally altered HCV specific CD4+ T cells in acute and chronic hepatitis C [100]. CD8+T cell exhaustion and persistent infection are more likely to develop when CD4+ T cells help is lacking or lost (Figure 2). Helper CD4+ T cells activate or license dendritic cells to optimally prime CD8+ T cells, recognition of antigen on the same antigen-presenting cell by CD4+ and CD8+ T cells is likely to be a key feature of antigen-specific T cell help. Thus the failure of CD4+HCV specific T cells may limit CD8+ T cells opportunities of priming by fully activated HCV antigen- loaded DC [27].

Another mechanism that may be involved in secondary T cell failure of HCV-specific CD4+ and CD8+ T cells in chronic HCV infection is signalling through programmed death 1 receptor (PD-1) (Figure 2). Down regulation of virus –specific T-cell responses via signalling through PD-1 on T cells has been linked with virus-specific T-cell deficiency during chronic viral infections in a murine model and in humans [101,102]. Several recent studies have demonstrated high expression levels of PD-1 in HCV-specific CD8+ T cells in patients with persistent HCV infection [55,95,103,104]. HCV-specific T cells that demonstrated increased expression of PD-1 on their surface exhibited impaired IFN-γ production, cytotoxic activity and proliferative potential in response to ex vivo HCV antigen stimulation [55,95,103]. Such impaired functional properties could be reversed by *in vitro* blockade of PD-1 interaction with its ligand PD-ligand 1 (PD-L1), demonstrating a causal relationship between PD-1 expression and exhaustion [95,103].

Different T-cell subsets with suppressive functions have been described (Figure 2). Among these, CD4+ CD25+ FoxP3+ regulatory T (Treg) cells have been involved in the control of auto-immunity and immune responses, through various mechanisms including the inhibition of APC maturation and T-cell activation (reviewed in [105]). An increased frequency of Treg cells has been observed in patients with chronic HCV infection compared to individuals who spontaneously resolved HCV infection [106–109]. However, a recent study in chimpanzees showed no difference in the frequency of Treg cells and the extent of suppression irrespective of the outcome of the infection [110]. Evidence against a role for Treg in promoting the development of chronic infection was recently reported in a prospective study of 27 acutely infected subjects. This study showed that there was no significant difference in the proportion of CD4+CD25^high^ T cells in the peripheral blood at baseline between the 15 subjects who developed chronic infection and the 12 subjects that subsequently cleared the infection [65]. The frequency for both groups was higher than in healthy controls and did not vary over time. Further studies are thus required to define the potential role of Treg in the outcome of primary HCV infection.

Viral escape from CD8+T cells is another important mechanism of T cell response failure in patients developing persistent infection [111–115] (Figure 2). Studies in humans and chimpanzees have shown that mutations in HLA class I restricted epitopes targeted by CD8+ T cells, occur early in HCV infection and are associated with persistence [116,117]. The role of HLA alleles in determining the outcome of HCV infection has been recently studied in an Irish cohort of women accidentally infected with HCV [118]. The HLA class I alleles A3, B27 and Cw*01 were associated with viral clearance whereas B8 was associated with viral persistence indicating that the host genetic background is an important variable that can influence infection outcome [118]. Interestingly stable cytotoxic T cell escape mutations have been linked to maintenance of viral fitness [119]. According to these authors, these observations elucidate potential mechanism by which viral persistence is established. Whereas consequences of stable integration of escape mutations into viral genomes are not clear, it is possible that epitopes presented by the most prevalent MHC class I molecules in human population will eventually be lost or become less dominant [120].

## 4. Conclusions and perspectives

In the last few years, considerable progress has been made in studying humoral and cellular responses in the course of HCV infection. While the role of neutralizing antibodies in outcome of HCV infection has long been questioned, the development of novel and convenient model systems for HCV infection showed an association between strong and early neutralizing responses and viral clearance. A self-limited course of acute hepatitis C is associated with a vigorous CD4+ and CD8+ T cell response targeting multiple HCV regions and with intrahepatic production of IFN-γ [23,24,51,56,57]. Clearance of HCV is thus probably mediated by a coordinated action of cellular and neutralizing immune responses. Only rare studies analyzed in parallel both humoral and cellular immune responses in the course of HCV infection [52]. Von Hahn *et al*. demonstrated that during chronic HCV infection in patient H, HCV is subjected to selection pressure from humoral and cellular immune responses resulting in the continuous generation of escape variants [52]. These data underscore that neutralizing antibody responses and cellular antiviral immunity are frequently impaired due to both viral and host factors leading to viral escape from the host’s immune surveillance and development of chronic infection.

Novel insights into the mechanisms underlying successful immune responses against HCV in individuals spontaneously clearing infection and elucidation of escape mechanisms from adaptive immune responses in chronic HCV patients will be essential for an improved understanding of HCV pathogenesis. Unravelling these important mechanisms of virus-host interactions will contribute to the development of novel strategies to prevent and control HCV infection.
